# Tropical forest soils serve as substantial and persistent methane sinks

**DOI:** 10.1038/s41598-019-51515-z

**Published:** 2019-11-14

**Authors:** Jun-Fu Zhao, Shu-Shi Peng, Meng-Ping Chen, Guan-Ze Wang, Yi-Bin Cui, Li-Guo Liao, Ji-Guang Feng, Biao Zhu, Wen-Jie Liu, Lian-Yan Yang, Zheng-Hong Tan

**Affiliations:** 1 School of Ecology and Environment, Hainan University, Haikou, 570228 China; 2 College of Urban and Environment Sciences, Peking University, Beijing, 100871 China

**Keywords:** Ecosystem ecology, Carbon cycle, Carbon cycle, Tropical ecology, Tropical ecology

## Abstract

Although tropical forest soils contributed substantially global soil methane uptake, observations on soil methane fluxes in tropical forests are still sparse, especially in Southeast Asia, leading to large uncertainty in the estimation of global soil methane uptake. Here, we conducted two-year (from Sep, 2016 to Sep, 2018) measurements of soil methane fluxes in a lowland tropical forest site in Hainan island, China. At this tropical forest site, soils were substantial methane sink, and average annual soil methane uptake was estimated at 2.00 kg CH_4_-C ha^−1^ yr^−1^. The seasonality of soil methane uptake showed strong methane uptake in the dry season (−1.00 nmol m^−2^ s^−1^) and almost neutral or weak soil methane uptake in the wet season (−0.24 nmol m^−2^ s^−1^). The peak soil methane uptake rate was observed as −1.43 nmol m^−2^ s^−1^ in February, 2018, the driest and coolest month during the past 24 months. Soil moisture was the dominant controller of methane fluxes, and could explain 94% seasonal variation of soil methane fluxes. Soil temperature could not enhance the explanation of seasonal variation of soil methane fluxes on the top of soil moisture. A positive relationship between soil methane uptake and soil respiration was also detected, which might indicate co-variation in activities of methanotroph and roots and/or microbes for soil heterotrophic respiration. Our study highlights that tropical forests in this region acted as a methane sink.

## Introduction

Methane (CH_4_) is an important greenhouse gas, and atmospheric methane concentration nearly tripled since 1750^1^, reaching 1857 ppb in 2018. The increase rate of atmospheric methane concentration is determined by the imbalance between methane sources and sinks. A comprehensive assessment of methane budget between 2000 and 2012 by Global Carbon Project shows a global total of ~550 Tg CH_4_ yr^−1^ emissions, with ~230 Tg CH_4_ yr^−1^ natural sources and ~320 Tg CH_4_ yr^−1^ anthropogenic sources respectively^2^. More than 90% of these methane sources are destructed by atmospheric chemical loss (hydroxyl radical, stratospheric loss and tropospheric Cl), and 5%-7% of these methane sources are consumed by methanotrophic bacteria in unsaturated oxic soil. Although soil methane uptake acts as an important methane sink, the global bottom-up estimation of soil methane uptake still has a large uncertainty of 9–47 Tg CH_4_ yr^−1 ^^2–4^.

Tropical forests act as methane sources^5^ is very rare. In contrast, tropical forest soil was overwhelmingly reported as important methane sinks^6^. Wet tropical forest swamps may contribute to the methane production^7,8^. Instead of methane sources, the large area of tropical forests standing on well-drained upland soils could be an important sink for atmospheric methane^9^. In the comprehensive meta-analysis of global field measurements of methane uptake^6^, tropical forest soils methane uptake (6.2 Tg CH_4_ yr^−1^) contributed to 28% of global soil methane uptake. This significant methane sink in tropical forest soils has the potential to change the atmospheric methane growth rate. However, compared to soil methane uptake studies in boreal and temperate forests, the sites with soil methane fluxes measurements in tropical forests are still sparse, especially in tropical Southeast Asia^10^. Whether tropical forest soils in Southeast Asia act as methane sources or sinks is still unknown. The monthly and annual magnitude, seasonal variation, and the drivers of methane fluxes in tropical forest soils in Southeast Asia are still elusive.

**Figure 1:**
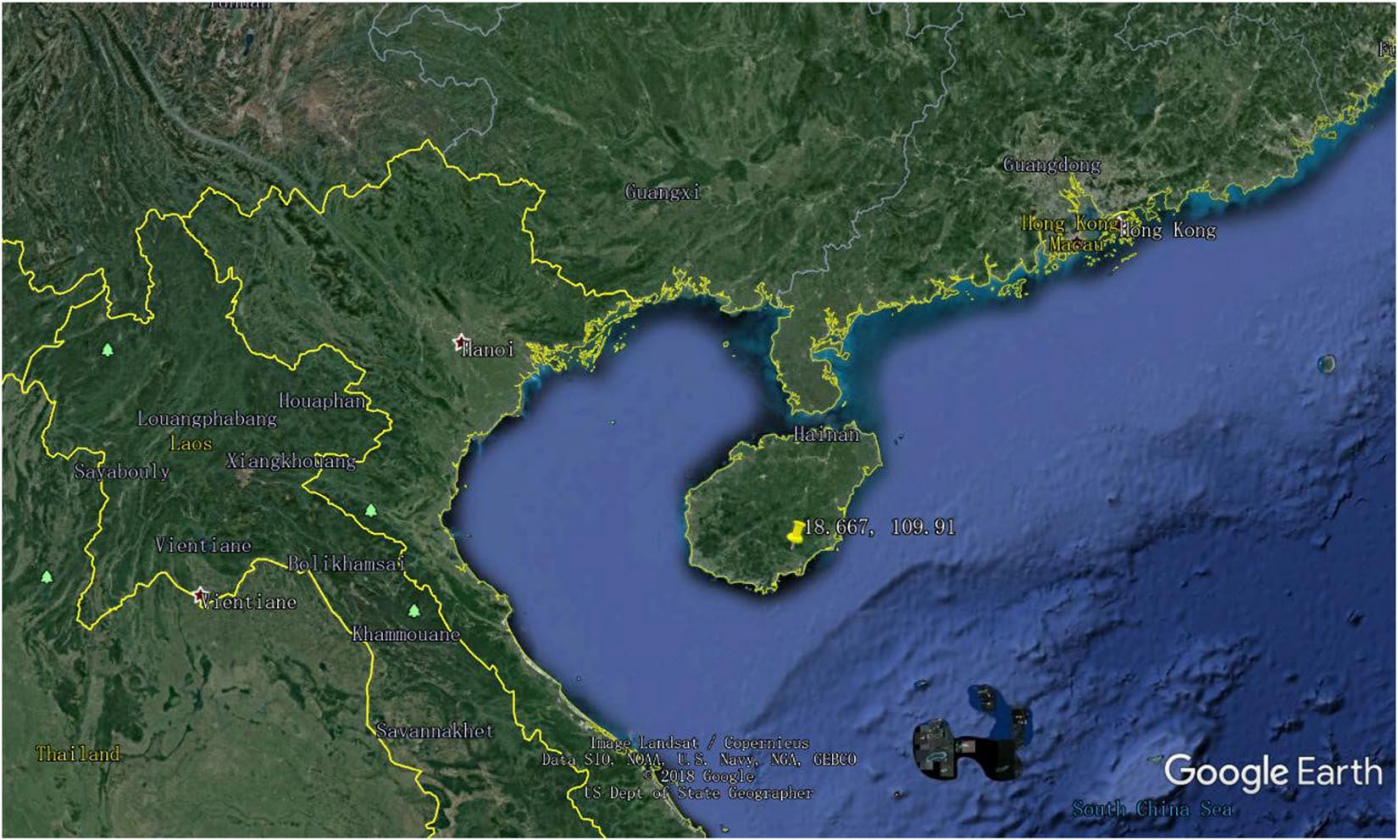
The geographic location of the research site.

To address the gap of methane fluxes in tropical forest soils in Southeast Asia, we started to measure soil methane fluxes since 2016 at a 1 ha plot established in a lowland tropical rainforest within Diaoluoshan National Nature Reserve in Hainan island (18°40′N, 109°54′E, elevation 255 m, Fig. 1). This plot is located on the northern edge of tropical Southeast Asia and is different from Neotropical forests near the equator in hydrothermal conditions. A total 15 collars were inserted into soils for methane and carbon dioxide fluxes measurements by a commercial greenhouse gas analyzer from LGR company (Model: 915-0011). With two consecutive years of soil methane fluxes measurements, our objectives were (1) to assess whether tropical forest soils at this study site were methane sources or sinks; (2) to explore the monthly and annual magnitude, seasonal variation in soil methane fluxes at this study site; and (3) to investigate the main drivers of soil methane fluxes at this study site.

## Results

### Soil climate

**Figure 2:**
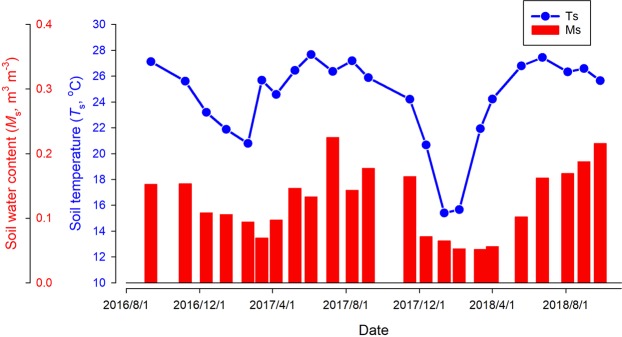
The variation on soil temperature (*T*s) and soil water content (*SWC*) during the observation period.

Both soil temperature (*T*_s_) and moisture (*M*_s_) were measured in each field campaign while simultaneously measuring methane flux. Figure 2 showed clear seasonal pattern in *T*_s_ and *M*_s_. As a typical East Asian monsoon climate here, warm temperature coincides with high moisture in the wet season, whereas cool temperature and low moisture level occurs in the dry season. The highest *T*_s_ occurs around June (~27.5 °C), and the lowest temperature occurs around January (~15.2 °C). Soil moisture in rainy season (June, July and August) is relatively high, but the lowest month of *M*_*s*_ in dry season occurs around March, with a minimum of about 0.05 m^3^ m^−3^. Note that nearly 4–5-month extreme cool and dry period (December 2017–April 2018) was observed.

### Temporal variations in soil methane flux

**Figure 3:**
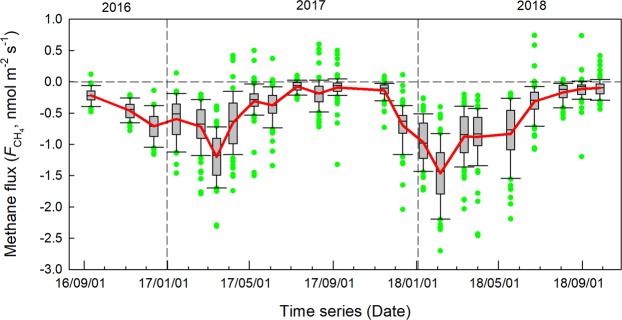
Temporal pattern on methane flux expressed as box plot. The red solid line connected the mean values.

Figure 3 showed the temporal variations in soil methane flux. The outliers represented by the green circles suggested that there were considerable spatial variations for each field campaign among the 15 collars within 1 ha plot. The mean value, connected by red solid line, was close to the median value shown in the box-plot, suggesting the mean value was a good statistical representative for each campaign. The mean soil methane flux showed that soils here served as persistent soil methane uptake, although there was a clear seasonality in this methane uptake, being stronger in the dry season and weaker in the wet season. The peak seasonal soil methane uptake occurred in February or March, more than −1.0 nmol m^−2^ s^−1^. The largest soil methane uptake during the two years (−1.43 nmol m^−2^ s^−1^) was observed in February 2018, although the lowest soil temperature was observed in February 2018 (Fig. 2). This extreme event and the seasonality of soil methane uptake may infer the control of soil moisture rather than soil temperature. Overall, by interpolating soil methane uptake from each campaign, annual methane uptake in 2017 (Sep. 2016 – Aug. 2017) and 2018 (Sep. 2017 – Aug. 2018) were estimated at 1.88 kg CH_4_-C ha^−1^ yr^−1^ and 2.12 kg CH_4_-C ha^−1^ yr^−1^ respectively. The average annual methane uptake was estimated at 2.00 kg CH_4_-C ha^−1^ yr^−1^.

### Soil moisture control over soil methane flux

**Figure 4:**
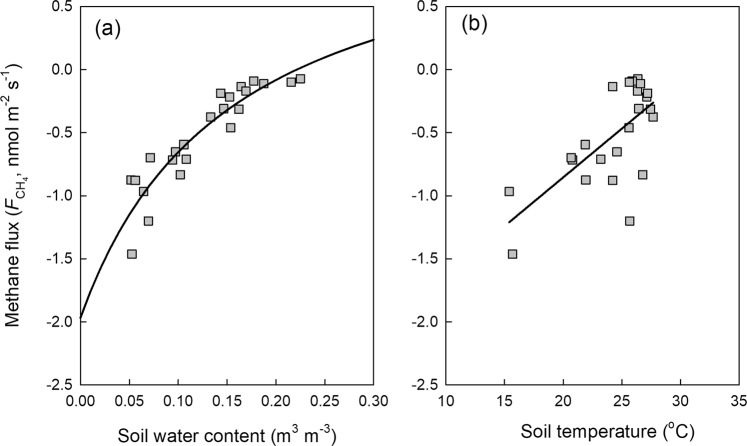
The dependence of methane flux on soil moisture (**a**) and temperature (**b**).

**Figure 5:**
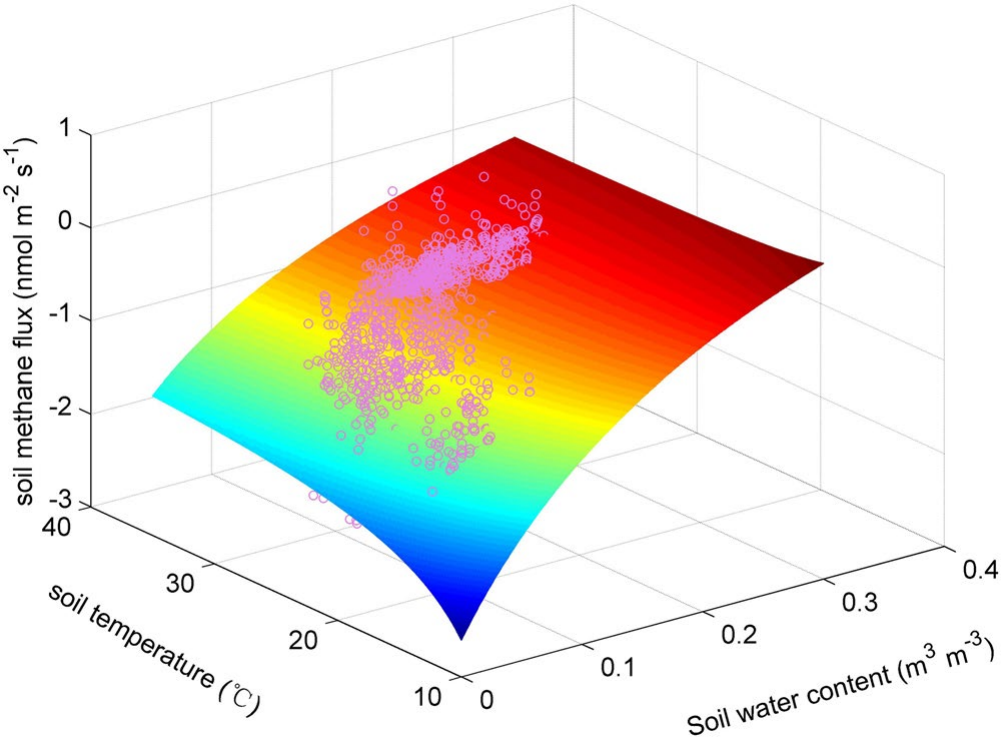
The co-control of soil moisture and temperature on methane flux.

Soil methane flux was highly dependent on soil moisture (Fig. 4a). Methane uptake (negative value indicates uptake) increased sharply when the soil was drying. Along with the increasing soil water content, methane uptake decreased continually and asymptotically approached zero when soil water content reached ~0.2 m^3^ m^−3^. When we used a modified rectangular hyperbola to fit the relationship between soil methane flux and environmental factors, the fitting *r*^2^ value was as high as 0.85 with a low RSME of 0.15 nmol m^−2^ s^−1^ (df = 22). Soil temperature was significantly and negatively correlated with soil methane flux (*r*^2^ = 0.47, RMSE = 0.28 nmol m^−2^ s^−1^). In principle, temperature would exponentially enhance biochemical reactions. The significantly negative correlation between soil temperature and soil methane flux could be due to the seasonal collinearity between soil temperature and moisture, given the fact that high temperature coincided with high water content under the monsoon climate (Fig. 2). In order to test whether soil temperature can enhance the *r*^2^ in prediction of soil methane flux by soil moisture, we used a bivariate model to simulate the methane fluxes observed in our study (Fig. 5). Little enhancement in *r*^2^ and RMSE was found by this bivariate model (*r*^2^ = 0.86, RSME = 0.14) compared to the fitting only with soil moisture (Fig. 4a). This confirmed that the main role of soil moisture rather than soil temperature in controlling the seasonal variation in soil methane flux.

### Relationship between soil methane and carbon dioxide fluxes

**Figure 6:**
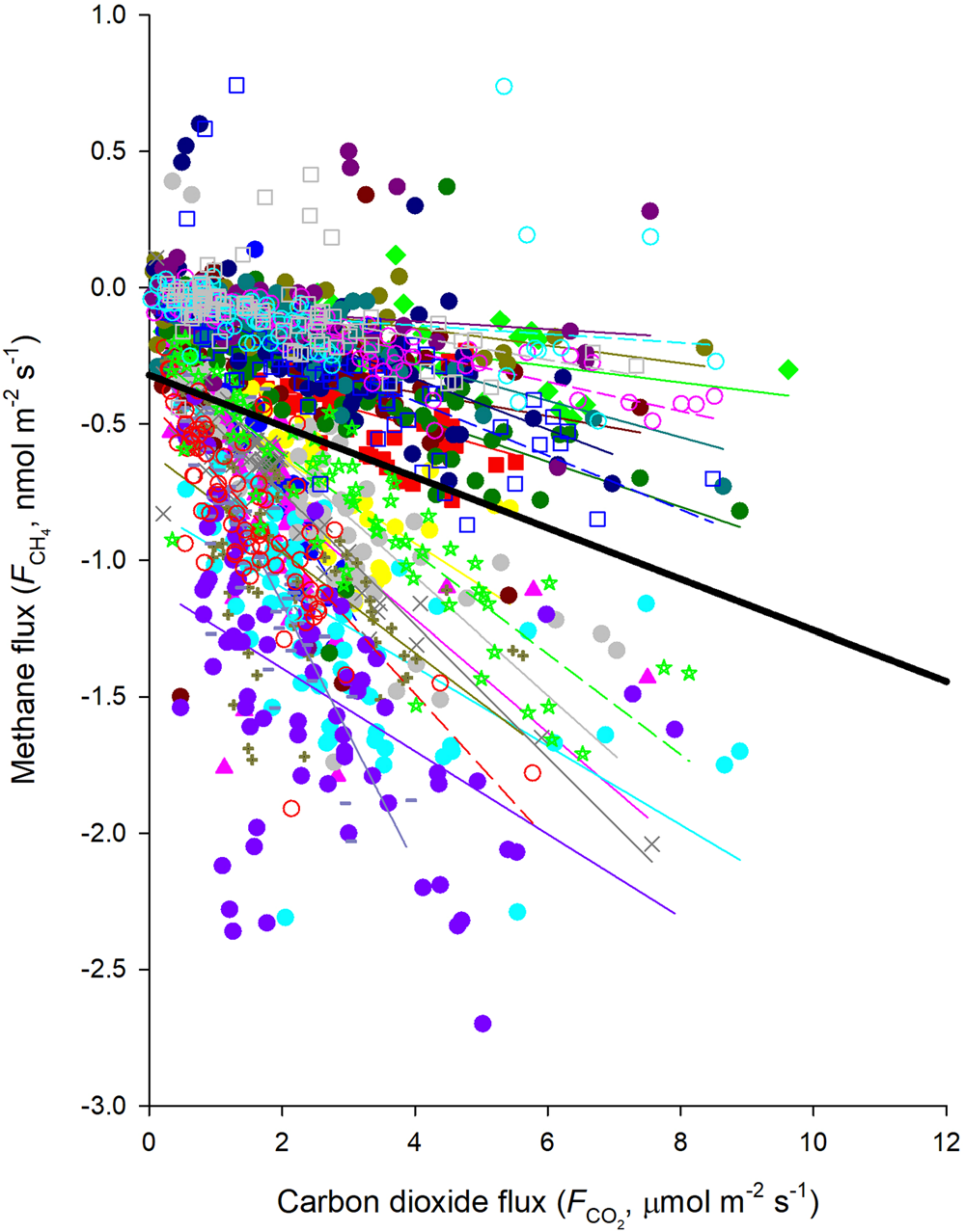
The relationship between soil respiration (${{\boldsymbol{F}}}_{{\bf{C}}{{\bf{O}}}_{2}}$) and soil methane flux (${{\boldsymbol{F}}}_{{\bf{C}}{{\bf{H}}}_{4}}$). Data points in different measurements were indicated with different colors. Lines represent linear regressions. The black bold line shows the linear regression with all data points.

**Figure 7:**
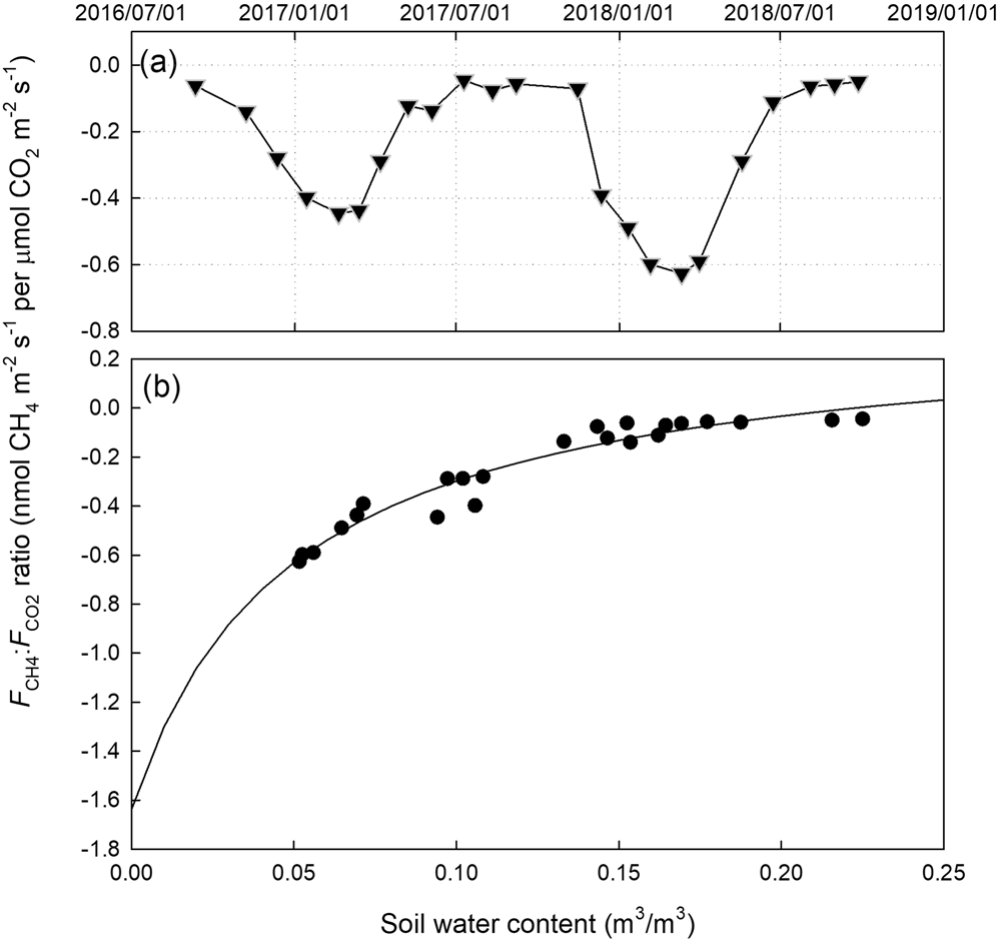
The variation of methane oxidation to soil respiration ratio (**a**) and the dependence of the ratio on soil moisture (**b**).

We found significant and negative correlations between soil methane flux (${F}_{{{\rm{CH}}}_{4}}$) and soil respiration (${F}_{{{\rm{CO}}}_{2}}$) across the 15 collars either for each field campaign or all data points together (Fig. 6). This indicated that soil methane uptake increased with larger soil respiration. The correlation coefficient and regression slope derived from the relationship between ${F}_{{{\rm{CH}}}_{4}}$ and ${F}_{{{\rm{CO}}}_{2}}$ showed clear seasonal patterns. The correlation was stronger and the slope was steeper in the dry season than those in the wet season. There was clear seasonality in the ${F}_{{{\rm{CH}}}_{4}}:{F}_{{{\rm{CO}}}_{2}}$ ratio (Fig. 7a). This ratio was higher in the dry season than that in the wet season. Similar to that of ${F}_{{{\rm{CH}}}_{4}}$, the ratio strongly depended on soil water content (*r*^2^ = 0.94, RSME = 0.049) (Fig. 7b).

## Discussion and Conclusions

### Tropical forest soils as substantial methane sinks

**Figure 8:**
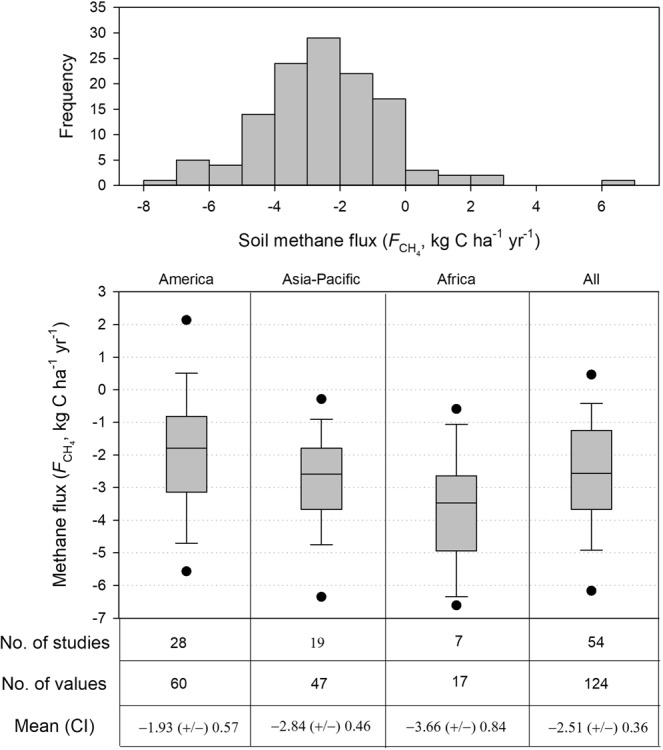
The literature review based annual methane flux estimates globally.

Based on two years of measurements, our results show that soil methane uptake was 2.00 kg CH_4_-C ha^−1^ per year at this tropical forests site. The magnitude of soil methane uptake at this site is within the previous reported range of tropical forest soils methane uptake (Fig. 8; Supplementary Table). We collected 124 estimates of soil methane fluxes in global tropical forests from 54 studies, and the tropical forest soil fluxes is estimated at −2.51 ± 0.36 (±95% confidential interval, CI) kg CH_4_-C ha^−1^ yr^−1^ (Fig. 8). Among the 124 estimates in tropical forests, 94% of soil methane fluxes show soil methane uptake in tropical forests. This indicates that tropical forest soils are substantial methane sinks. The mean tropical forests soil methane uptake in America, Asia-Pacific, and Africa are −1.93 ± 0.57, −2.84 ± 0.46, and −3.66 ± 0.84 kg CH_4_-C ha^−1^ yr^−1^, respectively. The geographic distribution of these estimates is uneven with nearly half of it obtained from Americas, and only 17 estimates were made in Africa. Our results of annual soil methane uptake is around 25% quantile of samples in tropical Asia-Pacific, i.e. lower soil methane uptake rate than other forest sites in tropical Asia-Pacific, and the reason may be the difference of hydrothermal conditions.

### Soil moisture as the dominant controller of soil methane fluxes

The soil methane sink is primarily contributed by biological oxidation. The key and first step for aerobic methanotrophs to oxidize methane could be expressed as^11^,

1 $${{\rm{CH}}}_{4}+{{\rm{O}}}_{2}+2{{\rm{H}}}^{+}+2{e}^{-}\mathop{\to }\limits^{{\rm{MMO}}}{{\rm{CH}}}_{3}{\rm{OH}}+{{\rm{H}}}_{2}{\rm{O}}$$

where MMO is an enzyme called methane monooxygenase. We could see from Eq. 4 that oxygen is indispensable for this enzymatic reaction. Consistent with most of the previous studies, soil moisture was the predominant controller of methane oxidation in our study site (Fig. 4 and Supplementary Table). More tropical forest soils methane uptake during the dry season than that during the wet season are observed at most tropical forests sites (Supplementary Table). Soil methane uptake was also observed to increase significantly during artificial^12,13^ or natural drought conditions^14^. The peak soil methane uptake in the driest and coolest month (Feb. 2018) during the two years at our study site also indicate that soil moisture dominates soil methane fluxes. When the methane oxidation reaction happen, both methane and oxygen must diffuse into soil where the methanotrophs are located. On the one hand, soil moisture could influence the diffusion rate of methane substrate and oxygen arriving to the oxidation zone through changing diffusion resistance and gas permeability^15–22^. Diffusion of gases in air is 10,000 times faster than their diffusion in water^23^. On the other hand, higher soil moisture with deeper depth of saturated soil layers could emit more emissions by methanogens, which offsets the upper soil layers methane uptake when net methane fluxes are measured at soil surface. These two mechanisms could explain why methane flux is highly sensitive to soil moisture. Different from the findings in the Harvard Forest soil^24^, we did not find moisture stress on methanotrophs even under very low soil moisture levels (Fig. 4). This may indicate that the soil methane sink will persist in the future even in the drier scenarios. The future drier climate may enhance methane uptake of the tropical forest soils due to higher atmospheric methane concentration and higher methane and oxygen diffusion rates into soil, but this depends on how drought stress impacts on the soil methanotrophs. If soil water content drop below wilting point, then the activities of soil methanotrophs will decrease.

As a biochemical reaction, temperature also plays a role in methane oxidation. Interestingly, compared to methanogens, methanotrophs seem to be less sensitive to temperature and have broader tolerance^20,23–25^. The optimum temperature for methane oxidation is 20 to 30 °C^26^. Numerous studies have shown no significant correlation between soil temperature and methane consumption^27–29^. In tropical regions, soil temperature seldom drops below 20°C, as well as soil temperature at our study site (Fig. 2). This implies that temperature plays a less significant role in controlling methane oxidation in tropical forest soils, which is supported by our results (Fig. 4 and Supplementary Table). Although we found a significantly negative relationship between soil temperature and ${F}_{C{H}_{4}}$ (Fig. 4b), it does not mean control of soil temperature on soil methane fluxes. This negative relationship between soil temperature and soil methane fluxes results from the co-variation between soil temperature and soil moisture in a monsoon climate. Therefore, we suggest that soil temperature plays a much less significant role in controlling methane oxidation in tropical forest soils.

It has been recognized that the main location for methane oxidation to occur is the uppermost mineral horizon of forest soil (oxidation zone)^19,30,31^. Verchot *et al*.^17^, claimed that consumption of atmospheric methane occurred in the superficial soil layers in the eastern Amazonia. In a pristine New Zealand forest soil, methanotrophic activity peaked at a depth of 5–10 cm^32^. Ishizuka *et al*.^33^, claimed that methane uptake rate depends on oxidation ability of the topsoil (0–5 cm) in the tropical forests of Southeast Asia. In a thick organic layer soil, highest methane uptake occurred at a depth of 5–10 cm^34^. Why the most active methane uptake zone does not exist in the surface soil organic horizon but in the subsoil could have two possible reasons. First, the competition for MMO caused by abundant ammonium in the organic horizon hampers the growth of methanotrophic bacteria. Second, for the moisture-sensitive methanotrophs, soil surface will experience much more water stress. Further work on soil methanotrophs is needed by incubating soils from different soil layers as well as process-based model to test the idea about vertical profile of methane oxidation.

Overall, our study supports that tropical forest soils in Hainan island serve as substantial methane sinks. The average annual methane uptake and carbon dioxide emission is estimated at 2.00 kg CH_4_-C ha^−1^ yr^−1^ and 9.08t CO_2_-C ha^−1^ yr^−1^. The seasonality of soil methane fluxes at our study site shows strong methane uptake in the dry season and weak methane uptake or neutral methane fluxes in the wet season. This study showed that soil moisture drove the methane flux in tropical forest soils of Hainan. We also found a significant positive relationship between methane uptake and soil respiration. The soil moisture can explain 94% of variation in the ratio between methane flux and soil respiration. This will help to estimate the soil methane fluxes at large scale. To date, comprehensive studies that simultaneously address ecosystem methane, and carbon dioxide exchange in the tropics are still scarce, particularly in China. Our study helps to better understand and quantify the impacts of climate change on ecosystem GHG budgets.

## Materials and Methods

### Study site

This study was carried out in Diaoluoshan national nature reserve of China. The national nature reserve is located in the south-eastern part of Hainan island, China (18°40′N, 109°54′E, elevation 255 m, Fig. 1). It was established in 2008 for the conservation of tropical rainforests. The climate shows strong seasonality with a predominant dry season starting from November through early April. The multi-year mean annual temperature is 24.6 °C, with the highest value occurring in July and the lowest in January^35^. Annual rainfall is high, up to 2160 mm with some of this total contributed by typhoons.

The studied forest could be categorized as a lowland tropical forest^36^, that mean canopy height is around 20–25 m and mean breast-height diameter is 9.67 cm. The canopy height is lower than that of inland tropical forests in the same latitude which might partly be a consequence of typhoons. The forest was logged before the national nature reserve was established. The studied forest is a about 40-year old stand with about 3,537 trees per hectare. The dominant species is *Vatica mangachapoi* which belongs to Dipterocarpaceae. *Dillenia turbinate, Gonocaryum lobbianum, Neolitsea obtusifolia, Antirhea chinensis, Psychotria rubra, Syzygium championii, Croton laevigatus, Phoebe tavoyana, Suregada glomerulata* were common species.

The soil type is krasnozem, developed from granite or igneous rock. The soil profile is clear with a mean soil depth deeper than 2.0 m. The soil properties of A-horizon are: 5.0 pH, 1.22% carbon, 0.11% nitrogen, 0.005% phosphorus, and 0.01% potassium^37^.

### Experiment design

The field campaign was conducted in a 1-ha permanent plot. All trees with diameter at breast height larger than 5 cm in the plot were labeled, censused, and identified to the species level. Three quadrats were selected in the permanent plot to install plastic collars for measuring soil methane and carbon dioxide fluxes. Five collars were installed in each quadrat, outer diameter 20 cm, length 10 cm. To facilitate the insertion into the soil, one end of the collar was inserted into the soil by 5 cm before use. Fieldwork began in September 2016 and continued until October 2018.

### Field measurements

We used a commercial greenhouse gas analyzer (GGA) to measure methane (${F}_{{{\rm{CH}}}_{4}}$) and carbon dioxide fluxes (${F}_{{{\rm{CO}}}_{2}}$). The portable GGA was purchased from Los Gatos Research Company (LGR Inc., San Jose, CAN). The GGA could store full absorption spectra for further processing or corrections on fluxes. We carried out the field measurements at monthly intervals. Soil temperature and moisture were measured simultaneously near the chambers at 0–5 cm depth with Decagon 5 TM sensor (Decagon Devices Inc., USA). We allowed enough time for the analyzer to warm up each time. We performed at least three measurements at each chamber. The measurements were conducted in the morning time before noon (8:00 am–12:00 am). We also tracked the diurnal variation of the fluxes. There was no clear diurnal pattern of methane fluxes and they varied only slightly in a day. Following the convention for flux measurement, we defined flux from the atmosphere into soil as negative, and vice versa.

### Statistics

We used Matlab 6.5.1 (Mathworks Inc., USA) for data analysis. The confidence interval was calculated as:

$${\rm{SE}}={\rm{std}}({\rm{x}})/{\rm{sqrt}}({\rm{lenght}}({\rm{x}}))$$

$${\rm{TS}}={\rm{tinv}}([0.025,0.0975],{\rm{length}}({\rm{x}})-1)$$

$${\rm{CI}}={\rm{mean}}({\rm{x}})+{\rm{TS}}\ast {\rm{SE}}$$

where SE is the standard error, TS is the T-Score, CI is the calculated confidence interval; std, sqrt, length, tinv, and mean are matlab functions.

The coefficient of determination (*r*^2^) and root mean squared error (RMSE) were calculated by a function contributed by Jered R Wells (file from Mathworks file exchange).

We found a hyperbola-like relationship and introduced the modified Michaelis-Menten equation^38^ to describe ${F}_{{{\rm{CH}}}_{4}}$ and ${F}_{{{\rm{CH}}}_{4}}/{F}_{{{\rm{CO}}}_{2}}$ ratio dependency on soil moisture,

2 $${\rm{y}}=\frac{{\epsilon }(x+b)}{{\epsilon }+x+b}$$

where $\epsilon$ and b are fitted parameters. Similar to other biochemical reactions, temperature dependency was described using Arrhenius equation^39,40^,

3 $${\rm{y}}={\rm{Aexp}}(-\frac{E}{8.31x})$$

where A and E are fitted parameters. We used the product of the two equations to show how temperature and moisture co-control methane fluxes,

4 $${\rm{y}}=\frac{{\epsilon }({M}_{s}+b)}{{\epsilon }+{M}_{s}+b}\,{\rm{Aexp}}(-\frac{E}{8.31{T}_{s}})$$

where *M*_s_ and *T*_s_ are soil moisture and temperature, respectively. The nonlinear fitting was conducted with the nlinfit command in Matlab 6.5.1 environment (Code and data are available through www.united-csfe.com/data/methane).

## Supplementary information

Supplementary Table

## Data Availability

The datasets generated and/or analyzed during the current study are available from the corresponding authors on request.
