# Supplementary material for: Tropical forest soils serve as substantial and persistent methane sinks
Source: Sci Rep. 2019 Nov 14;9:16799. doi: 10.1038/s41598-019-51515-z (PMC6856371; doi:10.1038/s41598-019-51515-z)
Supplement: Supplementary file 1 — Supplementary Table [file 41598_2019_51515_MOESM1_ESM.docx]

**TITLE PAGE**

**Title**

# Tropical forest soils serve as substantial and persistent methane sinks

Authors and their affiliation

Jun-Fu Zhao^1^, Shu-Shi Peng^2^, Meng-Ping Chen^1^, Guan-Ze Wang^1^, Yi-Bin Cui^1^, Li-Guo Liao^1^, Ji-Guang Feng^2^, Biao Zhu^2^, Wen-Jie Liu^1^, Lian-Yan Yang^1*^, Zheng-Hong Tan^1*^

^1^School of Ecology and Environment, Hainan University, Haikou 570228, China

^2^College of Urban and Environment Sciences, Peking University, Beijing 100871, China

*Corresponding authors:

Lian-Yan Yang ([yly@hainu.edu.cn](mailto:yly@hainu.edu.cn))

Zheng-Hong Tan ([tanzh@xtbg.ac.cn](mailto:tanzh@xtbg.ac.cn))

**Supplementary Table: Previous studies on Soil Methane Flux (**$\mathbf{F}_{\mathbf{CH}_{\mathbf{4}}}$**, kg CH4-C ha-1 yr-1) of Tropical Forests. N.A. Indicate Data not Available, WFPS is Water-Filled Pore Space.**

| No. | Country | $F_{{CH}_{4}}$ | Seasonality and control | Citation |
| --- | --- | --- | --- | --- |
| ***Americas*** | | | | |
| 1 | Brazil | -0.67, | N.A. | Keller *et al*.^1^ |
| 2 | Brazil | -0.79, 0.04, -1.29 | N.A. | Keller *et al*.^2^ |
| 3 | Ecuador | 1.13, -1.46 | N.A. | Keller *et al*.^2^ |
| 4 | Puerto Rico | -1.04, 2.72 | N.A. | Keller *et al*.^2^ |
| 5 | Panama | -2.13, -0.96 | N.A. | Keller *et al*.^3^ |
| 6 | Venezuela | -3.14 | N.A. | Scharffe *et al*.^4^ |
| 7 | Puerto Rico | -1.35 | N.A. | Steudler *et al*.^5^ |
| 8 | Costa Rico | -4.21, -4.37 | Higher in dry season, Soil diffusivity | Keller and Reiners^6^ |
| 9 | Brazil, | -4.44, -2.56 | Higher in dry season, WFPS | Steudler *et al*.^7^ |
| 10 | Brazil | -6.56, -6.98 | N.A., WFPS | dos Santos^8^; dos Santos *et al*.^9^ |
| 11 | Brazil | -4.76 | N.A. | Anderson and Poth^10^ |
| 12 | Costa Rico | -2.76 | N.A. | Weitz *et al*.^11^ |
| 13 | Brazil | -0.66, -2.41, -1.23, -2.85, -3.36 | N.A. | Goreau and de Mello^12^ |
| 14 | Brazil | -3.15, -2.95 | N.A. | MacDonald *et al*.^13^ |
| 15 | Brazil | -1.8, -1.57 | Higher in dry season, WFPS and respiration | Verchot *et al*.^14^ |
| 16 | Brazil | -0.83 | Higher in dry season, Soil water content | Fernandes *et al*.^15^ |
| 17 | Peru, | -2.62 | Higher in dry season, WFPS (not only diffusivity) | Palm *et al*.^16^ |
| 18 | Brazil | -0.83 | Higher in dry season, Soil water content | Davidson *et al*.^17^ |
| 19 | Brazil | -0.27, -0.82, -2.7, -2.46 | Higher in dry season, no dominant controller | Keller *et al*.^18^ |
| 20 | Brazil | -1.20 | Higher in dry season, soil water content | Davidson *et al*.^19^ |
| 21 | Brazil | -2.74, -4.90, -4.40 | Higher in dry season, WFPS | Sousa Neto *et al*.^20^ |
| 22 | Puerto Rico | -3.14, -1.37, -0.40 | Higher in dry season, soil water content | Wood and Silver^21^ |
| 23 | Ecuador | -1.06, -5.60, -3.10 | No clear seasonality, no dominant controller | Wolf *et al*.^22^ |
| 24 | Panama | -1.93, -0.37 | Higher in dry season, Nitrogen | Veldkamp *et al*.^23^ |
| 25 | Peru | -0.73, -4.01, -4.74 | Higher in dry season, WFPS | Jones *et al*.^24^ |
| 26 | Puerto Rico | -2.42, 0.56 | N.A., drought | O’Connell *et al*.^25^ |
| 27 | French Guiana | -3.67, 2.18, -1.77, -1.76, -0.95, 1.33 | Higher in dry season, soil water content | Courtois *et al*.^26^ |
| 28 | French Guiana | 6.43 | No clear seasonality, weak soil moisture control | Courtois *et al*.^27^ |
| ***Asia-Pacific*** | | | | |
| 1 | Malaysia | -3.68, -3.25, -2.56 | N.A. | MacDonald *et al*.^13^ |
| 2 | Indonesia | -1.85, -0.44, -1.11, -0.54 | No clear seasonality, WFPS not dominant | Ishizuka *et al*.^28^ |
| 3 | Australia | -2.4 | Higher in dry season, gas permeability | Kiese *et al*.^29^ |
| 4 | Australia | -2.08 | N.A., WFPS | Butterbach-Bahl *et al*^30^ |
| 5 | Indonesia | -3.32, -2.59, -4.64, -1.42, -1.79, -0.99, -3.18 | N.A., No dominant controller | Ishizuka *et al*.^31^ |
| 6 | Malaysia | 0.18 | No clear seasonality, no dominant controller | Melling *et al*.^32^ |
| 7 | China | -2.58 | N.A. | Werner *et al*.^33^ |
| 8 | China | -5.85, -4.14, -3.74 | No clear seasonality, no dominant controller | Tang *et al*.^34^ |
| 9 | Indonesia | -3.32, -3.10, -1.45 | No clear seasonality, no dominant controller | Purbopuspito *et al*.^35^ |
| 10 | China | -4.1, -3.1, -2.9 | Higher in dry season, soil water content | Yan *et al*.^36^ |
| 11 | Australia | -2.35, -2.41, -2.94 | Higher in dry season, WFPS | Kiese *et al*.^37^ |
| 12 | Malaysia | -2.94 | No distinct seasonality, no dominant controller | Yashiro *et al*.^38^ |
| 13 | China | -3.60, -2.50, -1.56 | High in the fall season, weak temperature dependency | Zhang *et al*.^39^ |
| 14 | China | -1.71, -3.86 | No clear seasonality, no dominant controller | Fang *et al*.^40^ |
| 15 | Malaysia | -1.34 | No distinct seasonality, soil water content | Itoh *et al*.^41, 42^ |
| 16 | Australia | -7.94, -2.46, -5.20 | Higher in dry season, WFPS | Rowlings et al.^43^ |
| 17 | Thailand | -6.68, -3.96, -2.41, -2.08 | Higher in dry season, no dominant controller | Vanitchung *et al*.^44^ |
| 18 | Indonesia | -3.63, -0.18 | Higher in dry season, nitrogen | Hassler *et al*.^45^ |
| 19 | China | -3.67 | No distinctive seasonality, no dominant controller | Wei *et al*.^46^ |
| ***Africa*** | | | | |
| 1 | Congo | -3.47, -0.59 | N.A. | Delmas *et al*.^47.^ |
| 2 | Congo | -5.28 | N.A. | Tathy *et al*.^48^ |
| 3 | Ghana | -2.56 | N.A. | Priemé and Christensen^49^ |
| 4 | Cameroon | -4.93, -3.22, -1.18 | N.A. | MacDonald *et al*.^13, 50^ |
| 5 | Kenya | -4.94 | N.A., Weak WFPS dependency | Werner *et al*.^51^ |
| 6 | Tanzania | -3.91, -2.73, -2.72 | No distinct seasonality, water | Gütlein *et al*.^52^ |
| 7 | Kenya | -3.16, -3.64, -2.42, -6.27, -6.61, -4.64 | Higher in dry season, WFPS | Wanyama *et al*.^53^ |

References

1. Keller, M., Goreau, T. J., Wofsy, S. C., Kaplan, W. A., & McElroy, M. B. Production of nitrous oxide and consumption of methane by forest soils. *Geophysical Research Letters*. **10**(12), 1156-1159 (1983).

2. Keller, M., Kaplan, W. A., & Wofsy, S. C. Emissions of N_2_O, CH_4_ and CO_2_ from tropical forest soils. *Journal of Geophysical Research: Atmospheres*, **91**(D11), 11791-11802 (1986).

3. Keller, M., Mitre, M. E., & Stallard, R. F. Consumption of atmospheric methane in soils of central Panama: effects of agricultural development. *Global Biogeochemical Cycles*. **4**(1), 21-27 (1990).

4. Scharffe, D., Hao, W. M., Donoso, L., Crutzen, P. J., & Sanhueza, E. Soil fluxes and atmospheric concentration of CO and CH_4_ in the northern part of the Guayana Shield, Venezuela. *Journal of Geophysical Research: Atmospheres*. **95**(D13), 22475-22480 (1990).

5. Steudler, P. A., Melillo, J. M., Bowden, R. D., Castro, M. S., & Lugo, A. E. The effects of natural and human disturbances on soil nitrogen dynamics and trace gas fluxes in a Puerto Rican wet forest. *Biotropica*. **23**(4), 356-363 (1991).

6. Keller, M., & Reiners, W. A. Soil‐atmosphere exchange of nitrous oxide, nitric oxide, and methane under secondary succession of pasture to forest in the Atlantic lowlands of Costa Rica. *Global Biogeochemical Cycles*. **8**(4), 399-409 (1994).

7. Steudler, P. A. *et al*. Consequence of forest-to-pasture conversion on CH_4_ fluxes in the Brazilian Amazon Basin. *Journal of Geophysical Research: Atmospheres.* **101**(D13), 18547-18554 (1996).

8. dos Santos, M. B. P. Medidas de fluxo de metano em solos de floresta da Mata Atlântica do Estado do Rio de Janeiro (Doctoral dissertation, PhD Thesis, Dept Geoquímica, Universidade Federal Fluminense Fluminense, Niterói). (1997).

9. dos Santos M.B.P., Nogueira S.R.A., Maddock J.E.L., & de Macedo J.R. Relationships between Simultaneous Methane, Nitrous Oxide and Carbon Dioxide Fluxes and Surface Soil Humidity and Temperature in the Mata Atlântica Subtropical Forest, Brazil. In: Drude de Lacerda L., Santelli R.E., Duursma E.K., Abrão J.J. (eds) Environmental Geochemistry in Tropical and Subtropical Environments. *Environmental Science*. (Springer, Berlin, Heidelberg, 2004)

10. Anderson, I, C., & Poth, M. A. Controls on fluxes of trace gases from Brazilian cerrado soils. *Journal of Environmental Quality*. **27**(5), 1117-1124 (1998).

11. Weitz, A. M., Veldkamp, E., Keller, M., Neff, J., & Crill, P. M. Nitrous oxide, nitric oxide, and methane fluxes from soils following clearing and burning of tropical secondary forest. *Journal of Geophysical Research: Atmospheres*. **103**(D21), 28047-28058 (1998).

12. Goreau, T. J., & de Mello, W. Z. Tropical deforestation: Some effects on atmospheric chemistry. *Ambio. Stockholm*. **17**(4), 275-281 (1988).

13. MacDonald, J. A. *et al*. The effect of termite biomass and anthropogenic disturbance on the CH_4_ budgets of tropical forests in Cameroon and Borneo. *Global change biology*. **5**(8), 869-879 (1999).

14. Verchot, L. V., Davidson, E. A., Cattânio, J. H., & Ackerman, I. L. Land-use change and biogeochemical controls of methane fluxes in soils of eastern Amazonia. *Ecosystems*. **3**(1), 41-56(2000).

15. Fernandes, S. A. P., Bernoux, M., Cerri, C. C., Feigl, B. J., & Piccolo, M. C. Seasonal variation of soil chemical properties and CO_2_ and CH_4_ fluxes in unfertilized and P-fertilized pastures in an Ultisol of the Brazilian Amazon. *Geoderma*. **107**(3-4), 227-241 (2002).

16. Palm, C. A. *et al*. Nitrous oxide and methane fluxes in six different land use systems in the Peruvian Amazon. *Global Biogeochemical Cycles*. **16**(4), 21-1 (2002).

17. Davidson, E. A., Ishida, F. Y., & Nepstad, D. C. Effects of an experimental drought on soil emissions of carbon dioxide, methane, nitrous oxide, and nitric oxide in a moist tropical forest. *Global Change Biology*. **10**(5), 718-730 (2004).

18. Keller, M. *et al*. Soil–atmosphere exchange of nitrous oxide, nitric oxide, methane, and carbon dioxide in logged and undisturbed forest in the Tapajos National Forest, Brazil. *Earth Interactions*. **9**(23), 1-28 (2005).

19. Davidson, E. A., Nepstad, D. C., Ishida, F. Y., & Brando, P. M. Effects of an experimental drought and recovery on soil emissions of carbon dioxide, methane, nitrous oxide, and nitric oxide in a moist tropical forest. *Global Change Biology*. **14**(11), 2582-2590 (2008).

20. Sousa Neto, E. *et al*. Soil-atmosphere exchange of nitrous oxide, methane and carbon dioxide in a gradient of elevation in the coastal Brazilian Atlantic forest. *Biogeosciences*. **8**(3), 733-742 (2011).

21. Wood, T. E., & Silver, W. L. Strong spatial variability in trace gasdynamics following experimental drought in a humid tropical forest. *Global Biogeochemical Cycles*. **26**, GB3005 (2012).

22. Wolf, K., Flessa, H., & Veldkamp, E. Atmospheric methane uptake by tropical montane forest soils and the contribution of organic layers. *Biogeochemistry*. **111**(1-3), 469-483 (2012).

23. Veldkamp, E., Koehler, B., & Corre, M. D. Indications of nitrogen-limited methane uptake in tropical forest soils. *Biogeosciences*. **10**(8), 5367-5379 (2013).

24. Jones, S. P. *et al*. Drivers of atmospheric methane uptake by montane forest soils in the southern Peruvian Andes. *Biogeosciences*. **13**(14): 4151-4165 (2016).

25. O’Connell, C. S., Ruan, L., & Silver, W. L. Drought drives rapid shifts in tropical rainforest soil biogeochemistry and greenhouse gas emissions. *Nature communications*. **9**(1), 1348 (2018).

26. Courtois, E. A. *et al*. Spatial Variation of Soil CO_2_, CH_4_ and N_2_O Fluxes Across Topographical Positions in Tropical Forests of the Guiana Shield. *Ecosystems*. **21**(7), 1445-1458 (2018).

27. Courtois, E. A. *et al*. Automatic high-frequency measurements of full soil greenhouse gas fluxes in a tropical forest. *Biogeosciences*. **16**(3), 785-796 (2019).

28. Ishizuka, S., Tsuruta, H., & Murdiyarso, D. An intensive field study on CO_2_, CH_4_, and N_2_O emissions from soils at four land‐use types in Sumatra, Indonesia. *Global Biogeochemical Cycles*. **16**(3), 22-1 (2002).

29. Kiese, R., Hewett, B., Graham, A., & Butterbach‐Bahl, K. Seasonal variability of N_2_O emissions and CH_4_ uptake by tropical rainforest soils of Queensland, Australia. *Global Biogeochemical Cycles*. **17**(2) (2003).

30. Butterbach-Bahl, K. *et al*. Temporal variations of fluxes of NO, NO_2_, N_2_O, CO_2_, and CH_4_ in a tropical rain forest ecosystem. *Global Biogeochemical Cycles*. **18**(3) (2004).

31. Ishizuka, S. *et al*. Spatial patterns of greenhouse gas emission in a tropical rainforest in Indonesia. *Nutrient Cycling in Agroecosystems*. **71**(1), 55-62 (2005).

32. Melling, L., Hatano, R., & Goh, K. J. Methane fluxes from three ecosystems in tropical peatland of Sarawak, Malaysia. *Soil Biology and Biochemistry*. **37**(8), 1445-1453 (2005).

33. Werner, C. *et al*. N_2_O, CH_4_ and CO_2_ emissions from seasonal tropical rainforests and a rubber plantation in Southwest China. *Plant and Soil*. **289**(1-2), 335-353 (2006).

34. Tang, X., Liu, S., Zhou, G., Zhang, D., & Zhou, C. Soil-atmospheric exchange of CO_2_, CH_4_, and N_2_O in three subtropical forest ecosystems in southern China. *Global Change Biology*. **12**(3), 546-560 (2006).

35. Purbopuspito, J., Veldkamp, E., Brumme, R., & Murdiyarso, D. Trace gas fluxes and nitrogen cycling along an elevation sequence of tropical montane forests in Central Sulawesi, Indonesia. *Global Biogeochemical Cycles*. **20**(3) (2006).

36. Yan, Y. *et al*. Fluxes of CH_4_ and N_2_O from soil under a tropical seasonal rain forest in Xishuangbanna, Southwest China. *Journal of Environmental Sciences*. **20**(2), 207-215 (2008).

37. Kiese, R., Wochele, S., & Butterbach-Bahl, K. Site specific and regional estimates of methane uptake by tropical rainforest soils in north eastern Australia. *Plant and Soil*. **309**(1-2), 211-226 (2008).

38. Yashiro, Y., Kadir, W. R., Okuda, T., & Koizumi, H. The effects of logging on soil greenhouse gas (CO_2_, CH_4_, N_2_O) flux in a tropical rain forest, Peninsular Malaysia. A*gricultural and Forest Meteorology*. **148**(5), 799-806 (2008).

39. Zhang, W. *et al*. Methane uptake responses to nitrogen deposition in three tropical forests in southern China. *Journal of Geophysical Research: Atmospheres*. **113**(D11), D11116 (2008).

40. Fang, H. J., Yu, G. R., Cao, M., & Zhou, M. Effects of multiple environmental factors on CO_2_ emission and CH_4_ uptake from old-growth forest soils. *Biogeosciences*. **7**(1), 395-407 (2010).

41. Itoh, M. *et al*. Temporal and spatial variations of soil carbon dioxide, methane, and nitrous oxide fluxes in a Southeast Asian tropical rainforest. *Biogeosciences Discussions*. **7**(5), 6847-6887 (2010).

42. Itoh, M. *et al*. Effects of soil water status on the spatial variation of carbon dioxide, methane and nitrous oxide fluxes in tropical rain-forest soils in Peninsular Malaysia. *Journal of Tropical Ecology*. **28**(6), 557-570 (2012).

43. Rowlings, D. W., Grace, P. R., Kiese, R., & Weier, K. L. Environmental factors controlling temporal and spatial variability in the soil‐atmosphere exchange of CO_2_, CH_4_ and N_2_O from an Australian subtropical rainforest. *Global Change Biology*. **18**(2), 726-738 (2012).

44. Vanitchung, S., Chidthaisong, A., & Conrad, R. Methane uptakes and emissions in upland tropical Forest and agricultural soils. *Journal of Sustainable Energy & Environment*. **5**, 43-49 (2014).

45. Hassler, E.*et al*. Soil fertility controls soil–atmosphere carbon dioxide and methane fluxes in a tropical landscape converted from lowland forest to rubber and oil palm plantations. *Biogeosciences*. **12**(19), 5831-5852 (2015).

46. Wei, H. *et al*. Variation in Soil Methane Fluxes and Comparison between Two Forests in China. *Forests*. **9**(4), 204 (2018).

47. Delmas, R. A., Servant, J., Tathy, J. P., Cros, B., & Labat, M. Sources and sinks of methane and carbon dioxide exchanges in mountain forest in equatorial Africa. *Journal of Geophysical Research: Atmospheres*. **97**(D6), 6169-6179 (1992).

48. Tathy, J. P. *et al*. Methane emission from flooded forest in Central Africa. *Journal of Geophysical Research: Atmospheres*. **97**(D6), 6159-6168 (1992).

49. Priemé, A., & Christensen, S. Methane uptake by a selection of soils in Ghana with different land use. *Journal of Geophysical Research: Atmospheres*. **104**(D19), 23617-23622 (1999).

50. Macdonald, J. A., Eggleton, P., Bignell, D. E., Forzi, F., & Fowler, D. Methane emission by termites and oxidation by soils, across a forest disturbance gradient in the Mbalmayo Forest Reserve, Cameroon. *Global Change Biology*. **4**(4), 409-418 (1998).

51. Werner, C., Kiese, R., & Butterbach‐Bahl, K. Soil‐atmosphere exchange of N_2_O, CH_4_, and CO_2_ and controlling environmental factors for tropical rain forest sites in western Kenya. *Journal of Geophysical Research: Atmospheres*. **112**(D03308) (2007).

52. Gütlein, A., Gerschlauer, F., Kikoti, I., & Kiese, R. Impacts of climate and land use on N_2_O and CH_4_ fluxes from tropical ecosystems in the Mt. Kilimanjaro region, Tanzania. *Global change biology*. **24**(3), 1239-1255 (2018).

53. Wanyama, I. *et al*. Soil carbon dioxide and methane fluxes from forests and other land use types in an African tropical montane region. *Biogeochemistry*. **143**: 171 (2019).
